# Axonal energy metabolism, and the effects in aging and neurodegenerative diseases

**DOI:** 10.1186/s13024-023-00634-3

**Published:** 2023-07-20

**Authors:** Sen Yang, Jung Hyun Park, Hui-Chen Lu

**Affiliations:** 1 The Linda and Jack Gill Center for Biomolecular Sciences, Indiana University, Bloomington, IN 47405 USA; 2 Department of Psychological and Brain Sciences, Indiana University, Bloomington, IN 47405 USA; 3 Program in Neuroscience, Indiana University, Bloomington, IN 47405 USA

**Keywords:** Aging, Axonopathy, Axonal bioenergetics, Energy metabolism, Glucose, Glycolysis, Mitochondria, NAD, Neurodegeneration, Neuroprotection

## Abstract

Human studies consistently identify bioenergetic maladaptations in brains upon aging and neurodegenerative disorders of aging (NDAs), such as Alzheimer’s disease, Parkinson’s disease, Huntington’s disease, and Amyotrophic lateral sclerosis. Glucose is the major brain fuel and glucose hypometabolism has been observed in brain regions vulnerable to aging and NDAs. Many neurodegenerative susceptible regions are in the topological central hub of the brain connectome, linked by densely interconnected long-range axons. Axons, key components of the connectome, have high metabolic needs to support neurotransmission and other essential activities. Long-range axons are particularly vulnerable to injury, neurotoxin exposure, protein stress, lysosomal dysfunction, etc. Axonopathy is often an early sign of neurodegeneration. Recent studies ascribe axonal maintenance failures to local bioenergetic dysregulation. With this review, we aim to stimulate research in exploring metabolically oriented neuroprotection strategies to enhance or normalize bioenergetics in NDA models. Here we start by summarizing evidence from human patients and animal models to reveal the correlation between glucose hypometabolism and connectomic disintegration upon aging/NDAs. To encourage mechanistic investigations on how axonal bioenergetic dysregulation occurs during aging/NDAs, we first review the current literature on axonal bioenergetics in distinct axonal subdomains: axon initial segments, myelinated axonal segments, and axonal arbors harboring pre-synaptic boutons. In each subdomain, we focus on the organization, activity-dependent regulation of the bioenergetic system, and external glial support. Second, we review the mechanisms regulating axonal nicotinamide adenine dinucleotide (NAD^+^) homeostasis, an essential molecule for energy metabolism processes, including NAD^+^ biosynthetic, recycling, and consuming pathways. Third, we highlight the innate metabolic vulnerability of the brain connectome and discuss its perturbation during aging and NDAs. As axonal bioenergetic deficits are developing into NDAs, especially in asymptomatic phase, they are likely exaggerated further by impaired NAD^+^ homeostasis, the high energetic cost of neural network hyperactivity, and glial pathology. Future research in interrogating the causal relationship between metabolic vulnerability, axonopathy, amyloid/tau pathology, and cognitive decline will provide fundamental knowledge for developing therapeutic interventions.

## Background

The mammalian brain is energetically demanding: consuming ~ 20% of the body’s total energy production despite accounting for only ~ 2% of body weight [1]. Glucose is the major brain fuel under normal circumstances [2, 3]. Thus, the brain mainly relies on glucose catabolism to generate adenosine triphosphate (ATP) and cannot afford any disruption of glucose and oxygen delivery [4]. Brain glucose uptake and catabolism to generate ATP have been extensively studied and reviewed [3–5]. Briefly, glucose metabolism requires glucose uptake into the brain cells, glycolysis in the cytoplasm and oxidative phosphorylation (OXPHO) in the mitochondria (Table 1). Each cycle of glycolysis yields 2 ATPs while OXPHO gives ~ 30 ATPs. In addition to pyruvate, neuronal mitochondria are capable of oxidizing ketone bodies and glutamine as alternative fuels when glucose and pyruvate availability is limited [6–9].

**Table 1:** Glucose metabolism

*Glucose transport*: Blood glucose is transferred across the blood–brain barrier through glucose transporter 1 (GLUT1) expressed in endothelial cells and astrocytes [10]. Then, glucose is transported into neurons through GLUT3 and GLUT4 [11–14], or imported into oligodendrocytes and microglia through GLUT1 [15, 16]
*Glycolysis*: Glucose undergoes glycolysis in the cytoplasm through a process consisting of 10 enzymatic reactions without oxygen involvement. The first five reaction steps are in the ATP investment phase, in which two molecules of ATP are used to metabolize one molecule of glucose. The remaining five reaction steps are the ATP payoff phase, through which two molecules of pyruvates and four molecules of ATP are generated. In net, two molecules of ATP are generated during each glycolysis cycle. Additionally, two molecules of NADH are generated from glycolysis and can be transported into mitochondria through the malate-aspartate shuttle for OXPHO
*TCA cycle and OXPHO*: Pyruvate derived from glycolysis is transported into mitochondria and undergoes oxidation through reactions in the TCA cycle. NADH and FADH_2_ are the product of the TCA cycle and serve to transfer electrons to feed the electron transport chain that drives ATP synthesis. About 30 molecules of ATP are generated in net from OXPHO per pyruvate.**]**

Recent human studies highlight bioenergetic maladaptation in brains upon aging and neurodegenerative disorders of aging (NDAs), such as Alzheimer’s disease (AD) [17], Parkinson’s disease (PD) [18], and Huntington’s disease (HD) [19]. Aging is considered one of the biggest risk factors for dementia and sporadic late-onset neurodegenerative diseases [20]. Single-cell transcriptomic analysis identified the decreased expression of mitochondrial OXPHO genes as a consistent signature of brain aging, prevalent across neurons and non-neuronal cell types [21, 22]. Meanwhile, proteomic and metabolomic profiling further demonstrate degraded mitochondrial metabolism in aged brains [23–25]. Primed by the aging process, neurodegenerative insults such as adverse environmental factors (e.g. unhealthy lifestyle [26], free radical or neurotoxicants exposures [27]), disease-associated genetic mutations [28], or epigenetic modifications [29] result in NDAs. During NDA progression, energy metabolism dysregulation is viciously aggravated along with functional decline [30]. Integrative bioinformatic analysis using brain multi-omics data has illuminated the age-dependent molecular and cellular changes in four major NDAs: AD [17], PD [18], HD [19] and amyotrophic lateral sclerosis (ALS) [31]. These studies consistently identify mitochondrial-related perturbations as a common pre-symptomatic feature.

To gain spatial information on glucose metabolism changes during aging/NDAs, numerous fluorodeoxyglucose-positron emission tomography (FDG-PET) studies have been conducted (selected studies listed in Table 2). A whole-brain metabolic network constructed from a large FDG-PET data set with subsequent diagnosis showed that the metabolic correlation between brain regions is progressively weakened as AD risks increase, and this disruption is more evident in females [32]. Glucose hypometabolism is often detected in AD-susceptible brain regions, which strongly predicts the incidence of mild cognitive impairment (MCI) in later life [33–37]. The majority of AD-susceptible regions identified are located in the default mode network (DMN), the topological central hub of the brain connectome [38, 39], containing densely interconnected long-range cortico-cortical axons [40, 41].

**Table 2:** Human neuroimaging studies

Disorders	Methods	Aims and Results	REF
MCI/AD	Imaging: FDG-PET for 66 AD, 23 early AD, 22 ctrl subjects	*Aimed to investigate cerebral glucose metabolism in early AD* **• Glucose uptake in PCC↓ MMSE scores↓**	[42]
	Imaging: FDG-PET and DTI with 20 early AD, 18 ctrl subjects	*Aimed to explore the association among hippocampal structural integrity, whole brain glucose metabolism and episodic memory with early AD subjects and ctrl* **• DTI diffusivity in anterior hippocampus↑ Glucose uptake in the anterior hippocampus, parahippocampal gyrus and the PCC↓ Episodic memory assessed by DVR↓**	[43]
	Imaging: FDG- and 11C-acetoacetate-PET for glucose and ketone metabolism with 51 MCI, 13 AD, 14 ctrl subjects	*Aimed to quantify both glucose and ketone metabolism in specific white matter fascicles associated with MCI and AD compared to ctrl* • **AD: Glucose uptake in the left posterior cingulate segment↓** • **AD: Ketone uptake in the left fornix and right parahippocampal segment of the cingulum↑**	[44]
	Imaging: MRI, FDG-PET, and PiB-PET for 40 noncarriers and 88 PSEN1, PSEN2, APP mutation carriers	*Aimed to use data from longitudinal study to identify pathophysiological biomarkers* **• Mutation carriers 10–15 yrs before AD: Bilateral hippocampal atrophy, Precuneus glucose uptake↓ Episodic memory↓**	[45]
	Imaging: fMRI, FDG-PET, and PiB-PET with 13 MCI (PiB +), 24 ctrl (12 PiB + and 12 PiB negative) older subjects	*Aimed to determine whether MCI elder individuals with increased amyloid burden have disruptions in the functional whole-brain connectivity in cortical hubs and if these disruptions are associated with dysfunctional glucose metabolism* **• Cortical hubs: Whole-brain connectivity↓ Glucose uptake↓**	[46]
	Imaging: FDG-PET of 12 relatives with APOE4, 19 relatives without APOE4, 7 AD subjects	*Aimed to determine if APOE4 is associated with brain function decline in relatives at risk for familial AD* **• APOE4 carriers at risk for AD: Parietal glucose uptake↓ Left–right metabolic asymmetry↑ • Dementia patients: Parietal glucose uptake↓↓**	[47]
	Imaging: FDG-PET and MRI with 11 AD and 54 non-demented subjects including 27 APOE4 and 27 non-carriers	*Aimed to find if the combination of cerebral metabolic rates and genetic risk factors can predict cognitive decline in AD* **• Non-demented APOE4 carriers: Glucose uptake in inferior parietal, lateral temporal, and posterior cingulate area↓ → (2 yrs later) Glucose uptake↓↓ Cognition↓**	[48]
	Imaging: FDG-PET and MRI of 11 APOE4 homozygotes, 22 ctrls without APOE4 allele	*Aimed to find whether the brain regions where glucose metabolism declines are also affected in subjects homozygous for the APOE4 allele before the onset of cognitive impairment* **• APOE4 homo carriers: Glucose uptake in the parietal, temporal, and prefrontal regions↓ PCC↓↓ Neuropsychological tests↓**	[49]
	Imaging: DTI with 61 ctrls, 56 MCI, 53 probable AD patients without a vascular component	*Aimed to report a comprehensive whole-brain study of diffusion tensor indices and probabilistic tractography obtained from healthy controls, MCI and probable AD subjects* • **Affected white matter in AD (vs ctrl): Cingulum bundle, the uncinate fasciculus, the entire corpus callosum and the superior longitudinal fasciculus** • **Affected white matter in MCI (vs ctrl): Crossing fibers in the centrum semiovale**	[50]
	Imaging: DTI with 63 autosomal-dominant AD PSEN1 & 2, or APP mutation carriers (32 asymptomatic and 31 symptomatic) and noncarriers (44 asymptomatic, 1 symptomatic)	*Aimed to identify the white matter pattern changes before detectable dementia in AD using early-onset autosomal-dominantly inherited AD subjects* **• AD mutation carriers 5–10 yrs before symptom: Structural integrity in posterior parietal and medial frontal regions of the white matter↓**	[51]
	Imaging: DTI, neurite orientation dispersion and density imaging (NODDI), q-space imaging with 40 cognitively normal ctrls, 38 subjective cognitive decline, and 22 MCI	*Aimed to use complementary diffusion metrics (i.e.,DTI, NODDI, and q-space) to study white matter alterations in early-stage AD* **Altered white matter tracts (cingulum, thalamic radiation, and forceps major) in MCI subjects:** • **Fractional anisotropy↓ (loss of fiber organization)** • **Radial diffusivity↑ (myelin degeneration or cell membrane deterioration)**	[52]
	Imaging: FDG-PET co-registered with T1-MRI (81 cognitively normal, 21 MCI, 15 AD); Louvain algorithm; Pearson correlation	*Aimed to determine if the strength of the brain metabolic network connectivity can predict the prognosis of MCI and AD and if it is modified by AD-risk gene expression* • **Subjects 5 yrs prior to AD diagnosis: Metabolic correlation between brain regions↓ (Female > male)** • **Expression of AD risk gene correlates with metabolic alteration in AD vulnerable regions**	[32]
PD	Imaging: FDG-PET and CT with 17ctrl and 23 PD subjects	*Aimed to evaluate the utility of the PD Related Pattern (PDRP) previously identified by FDG-PET and machine learning techniques, as a biomarker of early-stage PD* • **Glucose uptake in parieto-occipital and prefrontal regions↓** • **Glucose uptake in cerebellum, pons, thalamus, paracentral gyrus, and lentiform nucleus↑**	[53]
HD	Imaging: MRI and FDG-PET with 71 HD mutation carriers (24 pre-symptomatic and 47 symptomatic) and 30 ctrls	*Aimed to correlate anatomical and functional changes in various brain areas with the course of HD progression, estimated with a given expanded triplet number* • **Pre-symptomatic and symptomatic subjects: Gray- and white-matter volumes↓ Glucose uptake in frontal, temporal lobes, caudate and putamen↓** • **Pre-symptomatic: Progressive reduction of white matter**	[54]
FTLD/ALS	Imaging: FDG-PET with 22 C9ORF72 mutation carriers with FTLD, 22 non-carriers with FTLD, and 23 ctrls	*Previous MRI studies found changes in the thalamus and the cerebellum in C9ORF72-associated FTLD (C9FTLD). Here they aimed to examine functional changes* • **Mutation carriers: Glucose uptake in thalamus↓↓ Glucose uptake in frontal and temporal areas, cingulate cortex, Rolandic operculum, caudate nuclei↓** • **Non-carriers: Glucose uptake in right thalamus↓ Glucose uptake in frontal and temporal areas, right supplementary motor area, right supramarginal gyrus, right insula, right cingulate gyrus, right caudate nucleus, right postcentral gyrus, and right inferior parietal lobule↓**	[55]
	Imaging: FDG-PET with 32 ALS (13 with bulbar and 19 with spinal onset), 22 ctrls	• **Patients of both groups: Glucose uptake in the amygdalae, midbrain, pons, and cerebellum↑** • **Bulbar group (vs Spinal group): Glucose uptake in the large prefrontal and frontal regions↓ Neuropsychological tests score↓**	[56]

*Abbreviations*: *ALS* amyotrophic lateral sclerosis, *APOE* Apolipoprotein E, *APP* Amyloid precursor protein, *C9ORF72* chromosome 9 open reading frame 72, *ctrl* control, *DTI* diffusion tensor imaging, *DVR* Delayed verbal recall task, *FTLD* frontotemporal lobar degeneration, *MMSE* Mini Mental State Examination, *MRI* Magnetic resonance imaging, *PCC* posterior cingulate cortex, *PiB* Amyloid-beta biomarker, *PSEN1/2* presenilin1/2, *yrs* years

Compelling neuroimaging evidence from NDA patients further supports the prevalence of glucose hypometabolism in highly interconnected brain regions associated with functional impairment (Table 2). Briefly, human FDG-PET and diffusion tensor magnetic resonance imaging studies have revealed: (1) In MCI/AD patients, especially in AD-risk gene carriers, glucose hypometabolism preferentially occurs in DMN regions (such as posterior cingulate cortex, precuneus, anterior hippocampus and parahippocampal gyrus) [42–49], strongly correlated with white matter degeneration in these areas, and cognitive impairment [50–52]; (2) PD patients show hypometabolism in premotor and parieto-occipital cortex that correlates with motor dysfunction [53]; (3) HD patients show progressive glucose hypometabolism in the frontal lobe, temporal lobe, and striatum, accompanying white matter volume reduction [54]; (4) ALS patients exhibit complicated and region-diversified alterations in glucose metabolism, such as a decrease in prefrontal and frontal regions and an increase in hippocampus, parahippocampal gyrus and subcortical regions [55, 56].

NDA rodent models on isogenic backgrounds have been developed to gain mechanistic insights on NDA progression while controlling environmental factors. Resembling observations in human patients, dysregulated energy metabolism has been observed in the majority of NDA models starting at presymptomatic ages (Table 3). Using these animal models, significant molecular, cellular, and subcellular details at different stages of disease progressions have been acquired. Axonopathy is often observed simultaneously with bioenergetic impairment upon the onset of neurodegeneration (Table 3). Axons, the longest and the most morphologically complex subcellular compartment of most neurons [57–59], are particularly vulnerable to aging [60, 61], environmental insults [62] and neurodegenerative conditions [63–65]. Axonal activities require substantial energy supply [2, 66] and recent studies ascribe axonal maintenance failures to the dysregulation of axonal bioenergetics [67, 68]. Neurons cannot store high energy molecules, such as glycogen and fat, and thus they must synthesize ATP on demand [69, 70]. The latest high-resolution imaging studies reveal distinctive distributions and organization of mitochondria, the major ATP synthesizing organelle, in cell bodies, dendrites, and axons (Table 4) [71–75]. Surprisingly, despite the highly energetically demanding activities constantly taking place in axons, axonal mitochondria are not only sparsely distributed but are also smaller in size, have simpler morphology (Table 4), and show weaker metabolic activity compared to dendritic and somatic mitochondria [76]. In addition, axonal mitochondrial motility progressively decreases as neurons mature and age [77–80]. Notably, spatially confined mitochondrial compartments, consisting of single or multiple mitochondrial filaments with temporal stability of up to 80 min, are exclusively found in dendrites and soma, but not in axons [81]. This remarkable mismatch between the high energy demand and the less complex mitochondrial population in axons raises the following questions: *(1) How is bioenergetic machinery organized and regulated across axons to meet local energy needs? (2) How does aging, the major risk factor for neurodegeneration, affect axonal bioenergetics? (3) How does the aged axonal bioenergetic system go further awry as NDAs develop?*

**Table 3:** NDA rodent model studies (axon-related phenotypes are highlighted in bold)

Disease	Model		Hallmarks along disease progression		
AD	APP/PS1 (familiar APP and presenilin-1 mutaton)	Phenotypes	**3 M**: MCT 1, 2, 4↓ LDH-A↓ LDH-B↓↓ [82] OXPHO proteome change [83]	**6 M**: Carbohydrate metabolism↓ [84] **Detectable axonal pathology** [85]	**10 M**: Glucose flux into TCA cycle↓ [86] **Widespread axonal spheroids** [85, 87]
		Intervention	**6 M-9 M:** 15d-PGJ2 (PPARγ agonist) treatment → Glucose uptake↑ GLUT4↑ Spatial memory ↑ [88]		
			**7 M-8 M**: Albiflorin treatment → Mito dynamics↑ Antioxidant activity↑ Spatial memory↑ [89]		
			**9 M-10 M**: Electroacupuncture → Glucose uptake↑ GLUT1, 3↑ AMPK↑ Cognition↑ [90]		
			**9 M-23 M**: CP2 (mito complex I mild inhibitor) → AMPK↑Glucose uptake↑ Oxidative stress↓ Cognition↑ [91]		
	3xTg (familiar APP, PS1 and tau mutation)	Phenotypes	**P11-19**: Glycolysis↑ [92] Pentose phosphate pathway↓ [92] **1 M**: Mito biogenesis↓ [93]	**2 M**: Regional glucose uptake↓ [94] **Dystrophic axon** [95] **3 M**: PDH-E1α↓ Lipid peroxidation↑ [96] **Axonal transport↓** [97]	**6 M, 9 M**: COX IV↓Oxidative stress↑ [96] **12 M**: Mito respiration↓ [96] **18 M**: Whole brain glucose uptake↓ [94]
		Intervention	**3.5 M-18 M**: CP2 (mito complex I mild inhibitor) → Glucose homeostasis↑ Cognition↑ [98]		
			**6 M**: Intermittent hypoxic conditioning for 2wks → **8.5 M**: Mitochondrial bioenergetic↑ Learning and spatial memory↑ [99]		
	5XFAD (3 familiar APP and 2 familiar PS1 mutation)	Phenotypes	**2 M**: TCA cycle activity↓ [100] Synaptosomal glycolysis and OXPHO↓ [100] **Detectable axonopathy** [64] **3 M**: **Axonal lysosome accumulation** [101]	**4 M**:Antioxidant proteins↓ [102] Synaptosomal mito bioenergetics↓ [103] Synaptic ATP synthase subunit ↓ [104] **5 M**: **Peri-axonal Abeta thread** [105] **BACE1 accumulates in dystrophic axon** [106]	**7 M**: Glucose metabolism↓ (Female > Male) [107, 108] **8 M**: Neurotransmitter & glutamine↓ [109]
		Intervention	OSCP overexpression → **4-5 M** Mitochondrial function↑ Axonal mito dynamics and motility↑ Spatial learning and memory↑ [110]		
			**2 M-4 M**: Ergothioneine (antioxidant) treatment → Oxidative stress↓ Glucose metabolism↑ Fear memory↑ [111]		
			**4 M-6 M**: cx-DHED treatment → GLUT1,3↑ GLUT2↓ O-GlcNac level↑ Cognition↑ [112]		
			**4 M-6 M**: Liraglutide (GLP-1 analog) treatment → Glycolytic support from astrocyte↑ Spatial learning and memory↑ [113]		
PD	A53T α-Synuclein induction in vivo	Phenotypes	**4wks** post induction: Synaptic mito bioenergetics↓ [114] Altered distribution of glucose metabolism [115] **3-4wks** post induction: **Dystrophic axon** [116, 117]	**6wks** post induction: Mito inclusion and fragmentation [118]	**12wks** post induction: Perturbed TCA cyle and amino acid metabolism [119]
		Intervention	Mdivi-1 (Drp1 inhibitor) treatment prior to induction → **8wks** after: Synaptosomal mito bioenergetics↑ Motor function↑ [120]		
	A53T α-Synuclein induction in vivo	Phenotypes	α-Syn oligomerized at mito membrane → OXPHO complex I activity↓ROS↑mPTP opening [121] **Parkin mediated mitophagy in axon**↑ → Mito loss & Bioenergetic deficit [122] (Upon oxidative stress) Gene expression of OXPHO units↓Cholesterol synthesis↓Glycolysis↑Motor proteins↓ [123]		
			**Axon swelling Gene expression for axon guidance & synaptogenesis↓** [124] **Axon outgrowth↓ Axonal transport of vesicular cargos↓Autophagic flux↑** [125]		
	LRRK2 R1441G knockin in vivo	Phenotypes	**3 M**: Striatal synaptosomal OXPHO units↓ [126] **2-4 M**: **Axonopathy in SN** [127]	**12 M**: SN mito size↓ Mito fission↑ Autophagosome accumulation [128]	**18 M:** Mito ATP production↓ [129] **24 M**: Ubiquitinated mito↑ [129]
	LRRK2 G2019S mutation in vitro	Phenotypes	LRRK2 interaction with Drp1↑ → Drp1 recruitment to mito → Mito fragmentation & dysfunction [130] Mito DNA damage↑ [131] NAD^+^↓Sirtuin activity↓Mito motility↑ Mito respiration↓Mito density in distal neurite↓ [132] **Axonal transport of autophagosome**↓ [133]		
HD	R6/2 (Exon1 of human HD gene with 150 CAG repeats)	Phenotypes	**1 M:** Lactate↓ [134] **1.5 M**: Glucose uptake↓ [135]	**2 M**: **Axonal pathology in corpus callosum** [136] **2.5 M**: Oxidation of key metabolic enzymes [137] **Axon degeneration in sciatic nerve** [138]	**2-3 M**: Mito DNA damage↑ [139] **3 M**: Mito cristae integrity↓ [140]
		Intervention	**P21-3 M**: bezafibrate ( pan-PPAR agonist) treatment → Mito density↑ Oxidative stress↓ Motor function↑ [141]		
ALS	SOD1 G93A	Phenotypes	**P15**: **Axonal and NMJ mito length**↓ [142]	**P40**: Spinal cord mito respiration↓ [143] **P45**: **Axonal mito retrograde transport**↓ [142]	**3 M**: AMPK activation [143] **Mito clustering in sciatic nerve** [142] **Ventral root axon loss** [144]

*Abbreviations*: *AMPK* AMP-activated protein kinase, *BACE1* beta-secretase 1, *COX IV* Cytochrome c oxidase subunit 4, *cx-DHED* carboxy-dehydroevodiamine·HCl, *Drp1* Dynamin-related protein 1, *GLP-1* Glucagon-like peptide 1, *GLUT* glucose transporter, *LDH* lactate dehydrogenase, *LRRK2* Leucine-rich repeat kinase 2, *M* month, *MCT* monocarboxylic acid transporter, *Mito* mitochondria/mitochondrial, *mPTP* mitochondrial permeability transition pore, *NMJ* Neuromuscular junction, *OSCP* ATP synthase peripheral stalk subunit OSCP (ATP5PO)

**Table 4:** Differential distributions and characteristics of mitochondria (mito) in somata, dendrites, and axons

	**Species**	**Cell type/Brain region**	**Age**	**Soma**	**Dendrite**	**Axon**	**Reference**
% Area occupied by mito	Mouse	V1 L2/3 pyr. neurons	P36	N/A	> 50%	Distal axon, < 10%	[71]
	Mouse	L2/3 pyr. neurons	P21	N/A	69.58 ± 2.23%	8.41 ± 0.75%	[74]
	Mouse	L2/3 pyr. neurons	DIV21	N/A	69.6 ± 2.54%	4.95 ± 0.4%	[74]
	Rat	S1 L1-6	Juvenile	N/A	43.57 ± 1.73%	(Exc.) 11.19 ± 1.25%; (Inh.) 3.41 ± 0.65%; (Mye.) 0.40 ± 0.28%	[75]
Avg Volume	Mouse	V1 L2/3 pyr. neurons	P36	10–^2.5^-10^–0.5^ μm^3^	10^–2.5^—10^–0.5^ μm^3^	10^–2.5^—10^–1.5^ μm^3^	[71]
	Mouse	S1	Adult	N/A	0.89 ± 0.124 μm^3^	(Boutons) 0.056 ± 0.002 μm^3^	[72]
	Mouse	Hippocampal CA1 pyr. neurons	Adult	N/A	0.158 ± 0.017 μm^3^	(Boutons) 0.043 ± 0.002 μm^3^	[72]
	Mouse	Hippocampal CA1 pyr. neurons	4 m	~ 0.15 μm^3^	~ 0.14 μm^3^	(Mye.) ~ 0.27 μm^3^	[73]
	Mouse	Hippocampal DG granule neurons	4 m	~ 0.19 μm^3^	~ 0.27 μm^3^	(Mye.) ~ 0.12 μm^3^	[73]
	Mouse	Nucleus accumbens	11 m	N/A	0.195 ± 0.018 μm^3^	(Boutons) 0.05 ± 0.004 μm^3^	[72]
	Mouse	Dorsal cochlear nucleus	P17	N/A	1.357 ± 0.182 μm^3^	(Boutons) 0.375 ± 0.03 μm^3^	[72]
Mito length	Mouse	L2/3 pyr. neurons	P21	N/A	1.31–13.28 μm	0.45–1.13 μm	[74]
	Mouse	L2/3 pyr. neurons	DIV21	N/A	0.52 to 8.88 μm	0.3- 1.08 μm	[74]
	Mouse	V1 L2/3 pyr. neurons	P36	N/A	< 20 μm	< 1 μm	[71]
	Rat	Hippocampal neurons	DIV10	N/A	N/A	0.255–1.68 μm	[145]
Complexity index ^a^	Mouse	V1 L2/3 pyr. neurons	P36	< 25	< 55	< 10	[71]
	Mouse	Hippocampal DG granule neurons	4 m	~ 4.8	~ 6.9	(Mye.) 2.8	[73]
	Mouse	Hippocampal CA1 pyr. neurons	4 m	~ 4.7	~ 5.2	(Mye.) 3.5	[73]

*Abbreviations*: *avg* average, ~ , average, *DIV* days-in-vitro, *L* cortical layer, *m* month, *P* postnatal, *pyr*. Pyramidal, *V1* primary visual cortex, *S1* primary somatosensory cortex, *Exc* Excitatory, *Inh.* Inhibitory, *Mye.* myelinated

^a^ Complexity index is quantified as described in [146]

In this review, we specifically focus on central nervous system (CNS) axons, which can be divided into three subdomains: axonal initial segments, myelinated segments, and the axonal arbors harboring presynaptic boutons. We will first provide an overview of the subdomain-specific, energy-demanding events and characterize their locally specialized bioenergetic machineries; second, we will summarize the current literature on NAD redox homeostasis in axons, the mainstay of proper energetic metabolism; third, we will describe axonal bioenergetic maladaptation upon aging/NDAs and discuss how network hyperexcitability, NAD redox dysregulation, and pathological glial response could exacerbate axonal bioenergetic failures.

### Overview of energy-demanding events and bioenergetic machinery across distinct axonal subdomains

Mathematical modeling based on experimental data suggests energy consuming events vary by regions: in grey matter ~ 43% of energy expenditure is devoted to synaptic activities, while in white matter > 99% of energy is used for maintaining the resting membrane potential and other housekeeping tasks [2, 147, 148]. Among these expenditures, synaptic-related activities consume the highest amount of ATP [2]. As long-range axons in the brain travel through grey and white matter, their dominant energy consuming activities likely vary, because axonal regions in grey and white matter have different compositions of ion channels, organelles, biochemical machineries, and glial partners. Generally, CNS axons can be divided into three domains: (1) the axon initial segment (AIS), located adjacent to the soma and where action potentials originate, (2) the myelinated axon, an axonal shaft wrapped by layers of myelin sheath formed by oligodendrocytes, and (3) axonal collateral/terminal arbors, where axons form a plethora of *en passant* or terminal presynaptic boutons and are surrounded by astrocytes. Less ultrastructural and energetic data are available for lightly myelinated axons, abundant in the grey matter. For example, serotonergic, dopaminergic, and adrenergic axons which are barely myelinated [149, 150]. Due to a lack of data, we do not review the energetic regulation for these lightly myelinated axons here.

In the glia-neuron lactate shuttle hypothesis, neurons import lactate from astrocytes or oligodendrocytes through monocarboxylic acid transporters (MCTs), rather than directly taking up glucose for neuronal glycolysis. The lactate is then converted into pyruvate as the substrate for mitochondrial tricarboxylic acid (TCA) cycle and OXPHO [151, 152]. However, recent studies show that neuronal glycolysis is required for the ATP synthesis necessary to support neurotransmission and fast axonal transport [153–155]. In the neocortex, oligodendrocyte networks predominantly deliver glucose through MCTs to support compound action potentials in callosal myelinated axons [156]. Together, the above evidence suggests the existence of diverse glial metabolic support mechanismsin different axonal subdomains.

**Fig. 1:**
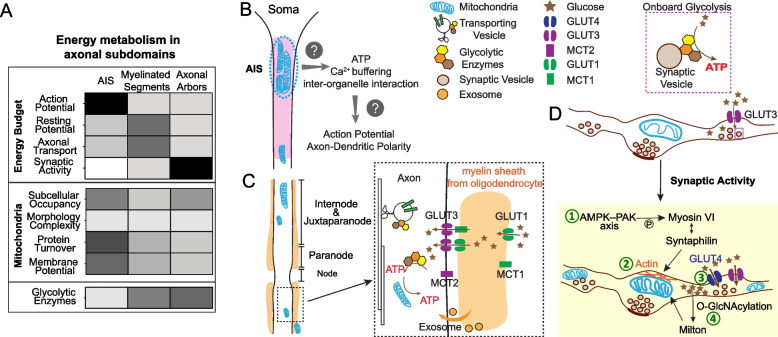
The spatial organization of glucose metabolism machineries across axon. **A**. Upper panel: Heat map description of the degree of energy consumption by each axonal activity within three axonal subdomains. Bottom panel: the intensity of each feature/parameter of glucose metabolism machineries within three axonal subdomains. Heat map color from white to dark represents the mild to strong degree/intensity. **B**.-**D**. The spatial organization of glucose transporters (GLUTs), monocarboxylic acid transporters (MCTs), glycolytic enzymes, mitochondria, and glial partners in axon initial segment (**B**), myelinated axonal shaft (**C**), and axonal arbors (**D**). **D**. The activity driven adaption of glucose metabolism machineries in presynaptic compartments. ① Activity dependent recruitment and anchoring of mitochondria to F actin filament at presynaptic sites through the AMPK-PAK-Syntaphilin axis; ② Neuronal stimulation induced mitochondrial ultrastructural changes with wider cristae and more compact or irregular matrices; ③ Surface mobilization of GLUT4, and presumably increased glucose uptake and glucose metabolism; and ④ Increased glucose metabolism through the Hexosamine synthesis pathway to boost O-GlcNAcylation of mitochondrial adaptor protein Milton, which reduce mitochondria mobility

Given that glucose metabolism is the dominant bioenergetic pathway for axons under normal dietary conditions, here we summarize the local energy demanding events that are primarily fueled by glucose metabolism and the spatial organization of the glucose metabolism machinery within each axonal subdomain (Fig. 1A). We hope the knowledge summarized here will provide a framework to delineate the sources of metabolic vulnerability of long-range axons.

#### Axon Initial Segment (AIS)

When the somatic membrane potential is sufficiently depolarized, an action potential (AP) is initiated in the AIS, the base of axon [157]. Mathematical modeling estimates the energy cost of AP propagation is ~ fourfold higher in grey matter than white matter [2] and the cost is highest at the AIS [158]. To form the focal point to initiate AP spikes, voltage gated sodium channels are densely anchored in the AIS to lower the voltage threshold for AP generation [159]. Subsequently, the highly amplified Na^+^ influx poses a high burden on the energy-consuming Na^+^/K^+^ ATPase to restore Na^+^ and K^+^ gradients. Studies looking at direct measurements of energy consumption during and after AP generation in the AIS remain to be conducted.

Stimulated emission depletion (STED) super resolution imaging and 3-dimensional (3D) electron microscopy (EM) reveal non-uniform mitochondrial coverage in axons. STED imaging of mouse cortical neurons and human induced pluripotent stem cells (iPSC) derived forebrain neurons shows that the proximal, rather than the distal region of the AIS retains an immobile cluster of mitochondria [160] (Fig. 1B). EM studies visualizing layer 2/3 pyramidal neurons of mouse primary visual cortex find that mitochondrial occupancy decreases as the axon extends further away from the soma [71]. Given that AP initiation likely dominates the energetic burden in AIS, it is tempting to ask if these clustered mitochondria are specifically recruited to provide copious ATP to fuel the efficient and accurate initiation and propagation of AP. Sensor imaging in the soma of hippocampal dentate granule neurons showed that increased Na^+^/K^+^ pump activity is the predominant activator of neuronal glycolysis upon neuronal stimulation [161]. Paradoxically, glycolytic enzymes are not enriched in the AIS, according to an AIS proteome profiling study [162], despite the enrichment of Na^+^/K^+^ pumps and its close proximity to the soma. Future studies are needed to determine the contribution of glycolysis to AIS energetics.

The AIS also plays a critical role in establishing and maintaining axon-dendrite polarity, in which the AIS actively guides selective and polarized protein trafficking to axons, and prevents protein diffusion from the somatodendritic area through the physical barrier formed by AIS-anchored membrane proteins and various cytoskeletal proteins [157]. Tau, a normally axonal microtubule-associated-protein, loses its polarity and is missorted in tauopathy conditions. Tjiang and Zempel recently demonstrated that locally restricted disruption of the membrane potential of mitochondrial clusters located in AIS results in Tau mis-sorting into soma, potentially due to defective microtubule dynamics [160]. It is unclear whether the polarized tau sorting demands substantial energy supplied by AIS-enriched mitochondria. It is also possible that clustered mitochondria provide Ca^2+^ buffering and/or the interactions with endoplasmic reticulum/endosomes to maintain the axon-dendrite polarity [163].

Supported by single axon EM reconstruction, proximal axons within ~ 100 µm of the soma lack myelination and oligodendroglial support [164]. Inhibitory axo-axonic synapses are selectively enriched onto the AIS region in excitatory pyramidal neurons [165, 166], while ~ 1% of AIS in cortex are associated with microglia [167]. It remains to be determined if glia-axon metabolic coupling exists in the AIS. Interestingly, ~ 75–85% of the AIS surface of rat and cat cerebellar Purkinje neurons are covered by glial processes [168]. The origin of these glial processes and their roles in axonal metabolism remain to be studied. It is also unclear whether extensive AIS glial coverage also occurs for other types of neurons.

#### Myelinated axonal segments

In mature CNS white matter, most axons are wrapped with a myelin sheath originating from oligodendrocytes. Myelination greatly improves the energetic efficiency of AP propagation, costing less than 1% of white matter’s energy budget [2, 169]. To achieve saltatory AP propagation, therefore improving the energetic efficiency of impulse propagation, CNS oligodendrocytes or peripheral nervous system (PNS) Schwann cells insulate most of the axonal surface with myelin sheath, rendering the nodes of Ranvier the only exposed areas where ion channels are densely embedded to allow AP regeneration. Due to their distinct structural and molecular organizations, the myelinated axonal segments can be further subdivided into nodes, paranodes, juxtaparanodes and internodes (Fig. 1C) [170]. Intriguingly, mitochondrial distribution and the external glial composition surrounding the nodes are drastically different between PNS and CNS axons. In PNS, upon Ca^2+^ elevation and Na^+^/K^+^ ATPase activation induced by electrical impulses, mitochondria are preferentially recruited to and accumulate in the nodes, likely providing on-site ATP synthesis to support rapid AP regenerations [171]. In CNS, mitochondria rarely distribute to the nodes but are present in the juxtaparanodes and internodes (Fig. 1C) [172–174]. However, a study in ex vivo cerebellar Purkinje cells shows that mitochondria reduce their motility in the nodes and paranodes in response to the potentiated electrical activity [174]. PNS nodes are solely contacted by the microvilli emanating from Schwann cells [170], while CNS nodes are mostly covered by astrocytic processes and sometimes contacted by oligodendrocyte precursor cells (OPCs) [175].

Within myelinated axonal segments, canonical synapses do not exist, and resting potential maintenance by plasma membrane Na^+^/K^+^ ATPase and various non-signaling (so-called ‘housekeeping’) tasks cost ~ 99% of the axon’s total energy budget [2]. Housekeeping activities comprise axonal transport, cytoskeleton rearrangement, protein/lipid synthesis, and mitochondrial proton leakage across the inner membrane [148, 176, 177]. Guedes-Dias P et al*.* [178] estimated that axonal transport costs less than 1% of the total energy budget for individual neurons. This estimate was based on the assumption that a single kinesin molecule is paired with a cargo and drives the transport process throughout. We hypothesize the energy cost for axonal transport to be much higher when one takes into account the complex molecular machineries involved in cargo-specific, dynamic, and bidirectional transport as well as the variety and large quantity of cargos. Axonal transport occurs in both retrograde and anterograde directions and includes the fast transport of synaptic vesicle precursors, RNA granules, endo-lysosome, autophagosome and mitochondria as well as the slow transport of cytoskeleton components and clathrin packets [179]. Evidence shows that even though kinesin and dynein drive transport in opposite directions, they attach to the same vesicular cargo through modulation by a variety of cargo-specific adaptors and coordinators [180]. The precise mechanism of kinesin and dynein’s cooperation was first explained by “the tug-of-war model”. In this model, the outcome of mechanical competition between kinesin and dynein determines the bidirectional transport behavior. This was, however, later challenged by “the paradox of co-dependence” phenomenon that inhibiting only one type of motors diminishes the motility in both directions [180]. Nevertheless, the exact energetic cost of axonal transport is hard to estimate without a detailed mechanistic understanding.

Emerging EM datasets allow us to compare mitochondrial morphology, size, and subcellular occupancy within the myelinated axonal compartment and unmyelinated *en passant* boutons (Table 5). In both subdomains, the mitochondria are similar in their morphological simplicity. However, myelinated regions are often occupied by fewer mitochondria with more heterogeneous sizes, including mitochondria of a larger volume than those seen in presynaptic compartments. It is unclear how this mitochondrial heterogeneity in myelinated axons develops and whether it has functional relevance.

**Table 5:** Mitochondrial comparison in myelinated and unmyelinated axon segments

	**Species**	**Brain region/Cell type**	**Age**	**Myelinated axon**	**Boutons**	**Reference**
Volume	Mouse	S1 cortex	Adult	N/A	0% ≥ 0.2 μm^3 ^~ 50% ≤ 0.05 μm^3^	[72]
	Mouse	Hippocampal CA1 pyr. neurons	4 m	~ 30% ≥ 0.2 μm^3^		[73]
	Mouse	Hippocampal CA1 pyr. neuron	Adult	N/A	0% ≥ 0.2 μm^3 ^~ 70% ≤ 0.05 μm^3^	[72]
	Mouse	Hippocampal DG granule neurons	4 m	~ 20% ≥ 0.2 μm^3^		[73]
	Mouse	Nucleus accumbens	11 m	N/A	0% ≥ 0.2 μm^3 ^~ 65% ≤ 0.05 μm^3^	[72]
	Mouse	Dorsal cochlear nucleus	P17	N/A	~ 45% ≥ 0.2 μm^3^	[72]
% Area occupied by mito	Rat	S1 L1-4	Juvenile	0.40±0.28 %	(Exc.) 11.19± 1.25%; (Inh.) 3.41±0.65%	[75]
	Mouse	S1	Adult	~ 2.87%	6.64% in average	[181]

*Abbreviations*: ~ , about, *L* cortical layer, *m* month, *P* postnatal, *pyr*. Pyramidal, *V1* primary visual cortex, *S1* primary somatosensory cortex, *Exc*. Excitatory, *Inh*. inhibitory

The sparse and heterogeneous distribution of mitochondria in myelinated axons raises the question on the energy source that fuels fast axonal transport, which is in constant demand to achieve fast and continuous movement of a large number of vesicular cargos in axons. Studies have shown that glycolytic enzymes are physically associated with fast-moving vesicles through their interaction with Huntingtin and provide onboard ATP synthesis to propel vesicular transport [154, 155] (Fig. 1D). Fast axonal transport in vitro is mainly supported by glycolysis, and OXPHO inhibition exerts a minimal impact on this form of transport [155]. The presence of the glycolytic complex on transporting cargos likely enables the efficient and continuous supply of ATP to overcome the lesser ATP efficiency of glycolysis compared to OXPHO. Future in vivo studies will be critical to determine whether glycolysis is the dominant energy source for fast axonal transport.

Using ex vivo acute brain slices, Meyer et al. showed that oligodendrocytes directly deliver glucose, instead of lactate, potentially through MCT1/2 and GLUT1/3, to axons in the corpus callosum (Fig. 1C), which indispensably supports the compound AP conduction [156]. Interestingly, GLUT1 incorporation into the oligodendroglial myelin compartment is upregulated upon optic nerve spiking activity [15]. It is currently unclear whether the enhancement of glucose uptake by neural activity, either directly through GLUTs on axons [14] or indirectly through oligodendrocytes [156], is a general mechanism to potentiate local glycolysis.

In addition to MCT-mediated metabolite exchange and activity-induced GLUT1 incorporation, Fruhbeis et al*.* shed light on the role of oligodendrocyte-secreted exosomes in sustaining axonal transport upon nutritional deprivation [182] (Fig. 1C). Exosomes can be secreted from diverse types of glia cells. It remains to be studied whether the glia-derived metabolic enzymes, metabolites and ATP can be delivered through exosomes to mediate the glia-axon metabolic coupling. Besides exosomes, a recent study showed that mitovesicles, a novel type of extracellular vesicles originating from mitochondria, are metabolically competent for autonomous ATP synthesis [183]. Proteome analysis suggests that neurons and astrocytes can secrete mitovesicles [183]. There is supporting evidence for the transfer of mitochondria from astrocytes to neurons to increase neuronal ATP level and viability upon cerebral ischemia in an astrocytic CD38-dependent manner [184]. Interestingly, damaged axonal mitochondria can also be released and then taken up by astrocytes for degradation. This has been observed in healthy adult optic axons and is implicated to occur similarly in superficial layers of cerebral cortex [185]. Future studies investigating the physiological roles of mitovesicles are likely to provide more insight into glia-axon metabolic coupling and whether such transcellular mitochondrial exchange takes place in axons as a non-cell autonomous mechanism for metabolic support and quality control.

#### Axonal arbors harboring pre-synaptic boutons

When axons enter their target zones, they often arborize extensively and form *en passant* or *terminal* presynaptic boutons full of synaptic vesicles (SVs) (Fig. 1D). These axonal arbors are mostly located in the grey matter and are rarely myelinated [186, 187]. The development of the genetically-encoded synaptic targeting ATP optical reporter *Syn-ATP* has enabled quantitative measurements of ATP alterations near SVs during physiological events to evaluate the local energetic costs at rest or during activation [188]. Pulido and Ryan [189] used the *Syn-ATP* reporter to show that the resting potential maintained by Na^+^/K^+^-ATPase only utilizes a minor portion of ATP in boutons [189], in contrast to what was previously believed [190]. Instead, they found that ~ 44% ATP is consumed by SV-resident vacuolar-type ATPase to restore the H^+^ gradient, which is constantly dissipated in resting state, despite the absence of SV exocytosis and recycling [189]. Upon electrical stimulation, ATP consumption in presynaptic boutons increases tremendously. During this active state, many calcium-driven processes and SV cycling are the major consumers of ATP, while Na^+^/K^+^ ATPase still consumes little [188]. Future studies should assess if these in vitro findings also occur in normal mature and aged brains and how NDA conditions impact them. In addition to SV cycling, it is plausible that local protein synthesis, occurring in ~ 40% of presynaptic terminals, also consumes a non-negligible amount of ATP [191] since adding one amino acid to the polypeptide chain requires ~ 2ATP and 2 guanosine triphosphate (GTP) [192], while proper protein folding by molecular chaperones also consumes ATP [193]. Furthermore, the robust anatomical plasticity of axons, reflected by the dynamic gain and loss of axonal arbors and presynaptic boutons, requires substantial protein synthesis as well as cytoskeleton remodeling [194]. Rearrangement of cytoskeletal components such as actin and microtubule occurs within minutes of electrical stimulation [195, 196] or long-term potentiation (LTP) [197]. Both actin and tubulin polymerization require ATP and GTP hydrolysis, respectively [148]. However, the degree of energetic burden posed by cytoskeleton remodeling remains to be determined.

Mitochondria are preferentially captured and stabilized at presynaptic boutons to serve as robust energetic factories [77, 198–201]. Still, less than 50% of boutons contain mitochondria [202–205], except lemniscal thalamocortical synapses where 92% of them contain mitochondria [206]. Facilitated by the creatine kinase/phosphocreatine system [207], ATP produced from mitochondria can rapidly diffuse over a certain range, therefore constantly fulfilling local bioenergetic needs in resident boutons, but also transiently compensating for the needs of nearby boutons lacking mitochondria [200, 208]. The dynamics and mobile behaviors of axonal mitochondria also impact energy metabolism. The molecular mechanisms governing these mitochondrial dynamics in neuronal subcellular compartments have been extensively reviewed [209–212] and thus we only highlight a few studies here.

In the *Drosophila* motor nerve terminal, the mitochondrial volume and densities within each bouton is positively correlated with the estimated energetic demand of each bouton [213], a correlation presumably caused by the activity-driven mechanism that couples energy consumption and synaptic mitochondrial recruitment. In mammalian neurons, several mechanisms governing synaptic mitochondrial positioning have been discovered. Chen et al*.* [214] showed that Ca^2+^ release triggered by action potentials binds to Miro, which then releases the C-terminal tail of KIF5 to bind syntaphilin (SNPH). Such KIF5–SNPH coupling then inhibits adenosine triphosphatase activity of kinesin-1, the molecular motor for mitochondria. In other words, SNPH immobilizes mitochondria at Ca^2+^ entry sites triggered by action potentials. SNPH also binds to dynein light chain LC8, which then enhances the docking between SNPH and microtubules to further reduce mitochondrial mobility [215]. Li et al*.* further showed that AMP-activated protein kinase–p21-activated kinase (AMPK-PAK) axis upregulates ATP production at presynapses in response to intense neuronal activity [216]. Upon prolonged electrical stimulation, the substantial energy usage in boutons activates AMPK-PAK axis, which then phosphorylates myosin VI to promote its interaction with SNPH (Fig. 1D). Subsequently, SNPH and myosin VI recruit and anchor mitochondria onto the presynaptic actin filament to support onsite ATP synthesis and Ca^2+^ buffering [216]. It has also been shown that GLUT4 is mobilized to the surface of axonal boutons in response to electrical stimuli and presumably increases local intracellular glucose concentration [14]. Elevated intracellular glucose promotes the O-linked-N-acetylglucosaminylation (O-GlcNAcylation) of the mitochondrial adaptor protein, Milton, which allows its interaction with four and a half LIM domains protein 2 (FHL2) to anchor mitochondria onto presynaptic actin filaments, therefore arresting mitochondria in the boutons [217, 218]. These arrested mitochondria can further adapt to a non-orthodox organellar configuration, featuring wider cristae and more compact or irregular matrices (Fig. 1D), according to an observation in mouse hippocampi upon LTP induction [219]. These presynaptic anchored mitochondria are considered to play important roles for higher order brain function, given the fact that the quantity of mitochondria per bouton in rhesus monkey’s prefrontal cortex positively correlates with working memory performance [220].

The findings of a positive correlation between the amount of oxidized/aged protein in mitochondrial matrix and the distance away from the soma suggest that mitochondrial protein quality control at distal axons is compromised [221, 222]. From a bioenergetic perspective, the mitochondrial membrane potential also declines in a distance-dependent manner from the soma both in vitro and in vivo [222]. Building on these observations, we speculate that mitochondria in the collateral arbor and terminal tips, far away from the soma, manifest compromised protein turnover and OXPHO capacity compared to peri-somatic mitochondria. EM data from neocortex and hippocampus showed that mitochondria are rarely present in dendritic spine heads or adjacent to the post-synaptic densities [71, 181, 205]. Thus, most mitochondria purified from synaptosomes are likely to arise from presynaptic compartments. Studies found that such presynaptic mitochondria are more susceptible to Ca^2+^ overload and damage induced by traumatic brain injury compared to non-synaptic mitochondria [223, 224]. Proteomic studies further reveal a differential proteome composition between synaptic and non-synaptic mitochondria [225–227]. However, the proteomics data should be interpreted with caution due to inconsistencies. For example, Stauch et al*.* showed most electron transport chain complex subunits are reduced in synaptic mitochondria including NADH:ubiquinone oxidoreductase core subunit S8 (NDUFS8) of complex I, ATP synthase F1 subunit alpha (ATP5A1) and ATP synthase F1 subunit beta (ATP5B) of complex V [225]. In contrast, these proteins were shown to be increased in synaptic mitochondria according to the findings from Volgyi et al*.* [226]. It is unclear whether the inconsistency was caused by mouse strain, age differences, or other variables in their studies.

Glycolysis, in parallel to mitochondrial OXPHO, contributes to ATP synthesis in presynaptic boutons during both resting and firing states [188, 189, 228]. In resting presynaptic boutons, the ATP level can be maintained for up to ~ 30 min by glycolysis alone when mitochondrial OXPHO is inhibited in vitro [188]. In autaptic hippocampal neurons, basal synaptic transmission can be fueled solely by glycolysis, while evoked transmission requires mitochondrial OXPHO [228]. In the calyx of Held, glycolysis is preferentially required to shape faithful AP waveforms and sustain synaptic transmission [229]. To cope with hypoxic stress, glycolytic enzymes are recruited to form a “metabolon” in synapses to sustain the synaptic function of serotonergic neurosecretory-motor neurons and preserve the locomotion behavior in *C. elegans* [230]. Although it is not known whether mammalian brains retain this “glycolytic metabolon” forming capability upon hypoxia, proteomics profiling shows the enrichment of glycolytic enzymes in striatal axon terminals of midbrain dopaminergic neurons in mouse brains [231]. The proteomic characterization of mammalian brain synaptic vesicles also identifies many glycolytic enzymes [232, 233] that physically associate with synaptic vesicles to fuel their transport along axons and their neurotransmitter uptake [154, 155, 234, 235]. Recent studies with cultured hippocampal neurons showed that presynaptic mitochondria possess the metabolic flexibility and capability to sustain synaptic transmission in the absence of glycolysis [236] when only oxidative fuel (lactate and pyruvate) was provided.

Glucose, the first substrate for the glycolytic pathway, is transported into neurons through the neuron-specific glucose transporters, GLUT3 and GLUT4 [11–14]. The majority of GLUT3 is embedded into the axonal plasma membrane and constantly uptakes glucose [14]. In contrast, only ~ 7.5% of GLUT4 is present on the axonal membrane at resting state, while the rest localizes to endosomal vesicles. In response to electrical stimuli, ∼20% of GLUT4, but not GLUT3, gets mobilized to the bouton surface (Fig. 1D) [14]. This additional GLUT4 likely increases glucose uptake and subsequently boosts glycolysis to support extra energy costs from synaptic activity. 6-Phosphofructo-2-kinase/fructose-2,6-bisphosphatase-3 (PFKFB3) is the master activator of glycolysis. However, its abundance in adult brain neurons remains low due to its constant degradation via the ubiquitin proteasomal pathway [237]. Intriguingly, juvenile hippocampus expresses higher levels of PFKFB3, which gets further upregulated during learning and is required for long-term memory formation exclusively in the juvenile period [238]. Along with the upregulation of GLUT3 and many other glycolytic enzymes [238], the increase in PFKFB3 likely potentiates glycolytic activity in juvenile hippocampus. However, evidence suggests that ubiquitous upregulation of PFKFB3 can be detrimental to mitochondrial function and neuronal health as it reduces pentose phosphate pathway flux and antioxidant capability [239, 240]. This raises the possibility that increased PFKFB3 in juvenile brain is exclusively restrained to synapses to boost local glycolysis without overtly interfering with the pentose phosphate pathway.

Presynaptic compartments are part of the “tripartite synapse”, in which the physical contact and functional integration of glial processes are an instrumental component [241]. 3D EM reconstruction of synapse-astrocyte contacts shows that more than 50% of presynaptic compartments are closely associated with astrocytes [242]. The metabolic coupling between astrocytes and neurons has been extensively studied and reviewed, mostly focusing on the lactate shuttle hypothesis [243–245], which has been questioned by stoichiometric and experimental evidence [246–248]. Some evidence suggest that lactate secreted from astrocytes modulates neuronal excitability as a signaling molecule by activating hydroxycarboxylic acid receptor 1 (HCAR1), a putative lactate receptor, in excitatory synapses [249]. It remains to be determined what other metabolites are shuttled from astrocytes to the presynaptic compartments in the “tripartite synapse” setting, whether MCTs or glia-derived exosomes are involved in this exchange, which metabolic pathways are mobilized, and how this metabolic coupling is fine-tuned by synaptic activity. The perspectives from Barros et al*.* suggest a putative coupling mechanism between astrocyte metabolism and synaptic activity through astrocytic Na^+^/K^+^ ATPases and Na^+^/HCO_3_^−^ cotransporters [250]. Astrocytes can sense the postsynaptic workload represented by the K^+^ efflux from postsynaptic compartment into the extracellular space upon the evoked excitatory postsynaptic potentials, then adapt their energy metabolism accordingly. Intriguingly, the recent discovery of the roles of endocannabinoids, a well-known retrograde messenger for synaptic transmission [251], in regulating mitochondrial metabolism in both neurons and astrocytes through mitochondrial type-1 cannabinoid receptor (mtCB_1_) [252, 253], suggests the potential involvement of the endocannabinoid system in homeostatic regulation of astrocyte-neuron metabolic coupling proportional to neural activity. Future studies will be required to confirm these activity-dependent astrocyte-neuron metabolic coupling hypotheses.

### NAD redox homeostasis underlying axonal bioenergetics

Nicotinamide adenine dinucleotide (NAD) is a critical cofactor mediating many redox reactions in glucose metabolism. The oxidized form of NAD (NAD^+^) acts as an electron acceptor while its reduced form (NADH) acts as an electron donor [254]. Pivotally, the NAD redox potential (NAD^+^/NADH) determines the thermodynamic driving force for the NAD^+^ or NADH consuming steps in both glycolysis and OXPHO [255]. Mechanisms involved in maintaining the NAD redox potential in mammalian cells include NAD biosynthesis, recycling, degradation, and subcellular compartmentalization and have been extensively reviewed [254, 256–258]. Here we focus on summarizing our current knowledge of NAD redox homeostasis in long-range axons.

#### NAD biosynthesis

**Fig. 2:**
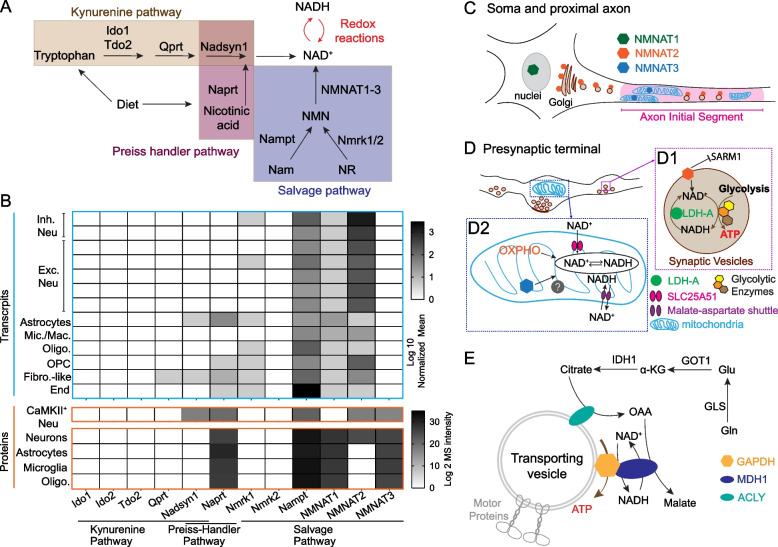
The molecular underpinnings of NAD redox potential maintenance in axons. **A**. A schematic view of NAD biosynthetic pathways. **B**. Heat map visualization of the abundance of mRNA and proteins of NAD biosynthetic enzymes. Transcript levels are adapted from [259]; protein levels are adapted from [260]. Abbreviations: Inh. Neu, inhibitory neurons; Exc. Neu, excitatory neurons; Mic. Mac., microglia and macrophages; Oligo, oligodendrocytes; OPC, oligodendrocyte precursor cells; Fibro.-like, fibroblast-like cells; End, endothelial cells. **C**. The subcellular localization of NMNAT1-3 in the soma and proximal axon. **D**. The subcellular distribution of NMNAT2, NMNAT3, and proteins involved in NAD^+^/NADH homeostasis in presynaptic boutons. (D1) NMNAT2, LDH-A, and glycolytic enzymes closely attach to synaptic vesicles; NMNAT2 inhibits SARM1 activation and maintains local NAD redox potential together with LDH-A to support “onboard” glycolysis; (D2) NMNAT3 inside mitochondrial matrix maintains local NAD redox potential together with OXPHO units, SLC25A51 (NAD^+^ transporter), and Malate-Aspartate shuttle. **E**. A hypothetical model of mitochondrial independent NAD^+^ recycling through the glutamine carboxylation pathway. MDH1, cytosolic malate dehydrogenase 1; ACLY, ATP citrate lyase; GLS, glutaminase; Gln, glutamine; Glu, glutamate; GOT1, glutamic-oxaloacetic transaminase 1; α-KG, alpha ketoglutarate; IDH1, isocitrate dehydrogenase (NADP^+^) 1; OAA, oxaloacetate

The salvage pathway is the major NAD biosynthetic pathway in the mammalian brain. All salvage pathway enzymes are present in the major brain cell types, while the enzymes involved in the kynurenine and Preiss-Handler pathways are minimally expressed in the brain (Fig. 2A-B; [259, 260]). In neurons, nicotinamide phosphoribosyl-transferase (NAMPT), the rate-limiting enzyme in the salvage pathway, is required for survival, mitochondrial homeostasis, and SV cycling [261–264]. NAMPT is localized to the cytoplasm and mitochondrial matrix in cortical neurons [265], suggesting the possibility that NAMPT in presynaptic mitochondria engages in local NAD biosynthesis to readily support activity-driven glucose metabolism. Notably, NAMPT can be exchanged transcellularly between cells through extracellular vesicles [266, 267]. Thus, axons can potentially receive additional NAMPT through extracellular vesicles secreted from their glial partners or from peripheral organs to boost local NAD^+^ production.

At the last step of the salvage pathway, nicotinamide mononucleotide adenylyl transferases (NMNAT) 1–3 catalyze NAD^+^ synthesis using nicotinamide mononucleotide (NMN) as their substrate. NMNAT1 is mainly localized in the nuclei (Fig. 2C) and is relatively uniformly expressed across different cell types [268, 269]. NMNAT2 is the most abundant NMNAT in the brain and is mainly expressed in neurons [269, 270]. Through palmitoylation, NMNAT2 associates with Golgi membranes and Golgi-derived vesicles including synaptic vesicle precursors in axons [271–273]. NMNAT2 is regarded as an axonal maintenance factor because its loss impairs axonal transport and results in axon degeneration in the absence of external insults [274–277]. Though NMNAT over-expressions can offer axonal protections [269, 278], studies also showed that excess amount of NMNAT can cause adverse consequence on presynaptic function in *Drosophila* [279] and visual circuit plasticity in mice [280].

Interestingly, OPCs, the unique glia cells forming synapse-like contacts with axons, abundantly express NMNAT2 mRNA [281, 282]. It is unknown whether OPC-NMNAT2 is involved in OPC-axon metabolic coupling. Knowing that OPCs secrete exosomes [283], it will be interesting to investigate whether membrane-bound NMNAT2 can reach axons through OPC-derived exosomes and facilitate axonal NAD biosynthesis.

The literature suggests that NMNAT3 is a mitochondrial-targeted isoform [270, 284]. Despite extremely low NMNAT3 mRNA levels in mouse brain, NMNAT3 protein has been detected in cultured cortical neurons [265], adult mouse brain [260], and human postmortem brain [285]. Future study is required to further confirm NMNAT3’s existence as well as to investigate its physiological function in neurons.

#### NAD^+^ recycling from NADH in cytoplasm

In addition to de novo NAD biosynthesis, the NAD redox potential can be replenished via NAD^+^ recycling from NADH derived from glycolysis and the TCA cycle (Fig. 2D). Based on pharmacological and genetic studies, mitochondrial OXPHO seems to be the major NAD^+^ recycling pathway in neurons. NAD^+^ is regenerated from NADH in the mitochondrial matrix through mitochondrial respiratory complex I (NADH-ubiquinone oxidoreductase) and then exchanged to the cytoplasm NAD^+^ pool through the malate-aspartate shuttle [286–288]. However, the low density and immobility of axonal mitochondria in many axonal regions (see above) pose significant challenges for mitochondrial regeneration of NAD^+^ to restore the axonal NAD redox potential. Knowing that glycolysis supplies ATP in the absence of mitochondria in some axonal segments, mitochondria-independent NAD^+^ recycling mechanisms are likely to be present in those axonal regions to restore the NAD redox potential. Future studies will be required to test this hypothesis.

One type of mitochondria-independent NAD^+^ recycling is mediated by lactate dehydrogenase isoform A (LDH-A) that is associated to the fast-moving axonal transport vesicles (Fig. 2D). LDH-A promotes the conversion of pyruvate to lactate. During this enzymatic reaction, it regenerates NAD^+^ by oxidizing glycolysis derived NADH and therefore facilitates the vesicular glycolysis that is required for fast axonal transport [289, 290]. However, if lactate gets exported from glia to axons following the proposed lactate shuttle hypothesis, lactate dehydrogenase isoform B (LDH-B) will be required to convert lactate into pyruvate for subsequent mitochondrial oxidation. Because LDH-B favors the conversion in reverse direction to LDH-A, that is it consumes NAD^+^ and produces NADH and pyruvate [291, 292], LDH-B's activity may counteract LDH-A, thus reducing NAD redox potential and restraining glycolysis.

Gaude et al*.* employed a mammalian cell model of tunable mitochondrial dysfunction to shut down mitochondrial-dependent NAD^+^ recycling and uncovered that the cytosolic reductive carboxylation of glutamine as an alternative route for NAD^+^ recycling [293]. The glutamine reductive carboxylation takes over the mitochondrial TCA cycle to provide oxaloacetate as the substrate for malate dehydrogenase 1 (MDH1), which subsequently regenerates NAD^+^ from NADH and physically couples with GAPDH to facilitate glycolysis (Fig. 2E) [293]. It has also been shown that cultured cortical neurons possess metabolic flexibility to oxidize glutamine when mitochondrial pyruvate uptake is inhibited [9]. The enzymes involved in glutamine carboxylation, such as glutaminase (GLS), glutamic-oxaloacetic transaminase 1 (GOT1), glutamic-oxaloacetic transaminase 2 (GOT2), isocitrate dehydrogenase (NADP^+^) 1 (IDH1), ATP citrate lyase (ACLY) and MDH1, are highly expressed in neurons (Fig. 2E, [259, 260] and Allen brain single cell transcriptomics explorer). Of note, ACLY is shown to be enriched in vesicles for axonal transports [294]. Whether and in what context glutamine carboxylation contributes to axonal NAD redox homeostasis merits further investigation.

#### Discrete NAD^+^ pool in mitochondria

NAD redox potential in mitochondrial matrix is typically ~ 10–100 fold lower than the potential in the cytoplasm due to the active NADH import through malate-aspartate shuttle [295]. However, the absolute NAD^+^ pool within mitochondria account for ~ 50–70% of total cellular NAD^+^ and exhibits relatively long half-life in contrast to the labile cytosolic NAD^+^, based on NAD sensor studies in mammalian cell lines [296–299]. The mitochondrial NAD^+^ transporter, SLC25A51, may be responsible for maintaining such discrete NAD^+^ pool by actively importing NAD^+^ from the cytoplasm [300–302]. In addition to NAD^+^ import, NAD synthesis through the Salvage pathway enzymes, NAMPT and NMNAT3, residing inside mitochondria in certain cell types [303, 304], can further increase mitochondrial NAD^+^. In neurons, the quantity and stability of mitochondrial NAD^+^ pool across various axonal subdomains have not been determined. These measurements are particularly important for presynaptic boutons where mitochondria support the intense synaptic activities while enduring the potentially incompetent protein turnover and Ca^2+^ buffering [222, 223]. Single cell transcriptomic and proteomic data suggest the expressions of SLC25A51 and NMNAT3 in neurons (Allen brain single cell transcriptomics explorer and [260]). Future studies are required to elucidate their roles in mitochondrial NAD^+^ pool maintenance, TCA cycle and OXPHO as well as their requirement for axonal functions.

#### NAD consuming pathways

Besides glucose metabolism, the fluctuations of NAD^+^ and its metabolites can elicit a profound impact through NAD^+^ consuming enzymes, such as Sirtuins (SIRTs) and Poly (adenosine diphosphate-ribose) polymerases (PARPs) [305–307]. SIRTs are well known in governing mitochondrial biology and metabolic homeostasis [305, 308, 309]. Noteworthy, Chamberlain et al*.* discovered a novel form of glia-axon metabolic coupling through SIRT2 [310]. They found that oligodendrocytes provide SIRT2 to myelinated axons through exosomes. Upon reaching axons, SIRT2 deacetylates mitochondrial adenine nucleotide translocase 1/2 and enhances mitochondrial ATP production. PARPs play diverse roles in mRNA processing, ribosomal biogenesis, and protein ubiquitination [311]. It is unclear whether PARPs are present in axons.

Sterile alpha and TIR motif containing protein 1 (SARM1) catalyzes NAD(P) hydrolysis and is a central regulator of an axon-self destruction cascade [312–315]. Distinct from SIRTs and PARPs, SARM1 is a multifaceted metabolic sensor detecting changes in NAD^+^, NADP^+^ and NMN levels. Under normal physiological conditions, when NAD^+^ and NADP^+^ levels are actively maintained by NMNAT2 and NAD^+^ kinase (NADK) [268, 316], SARM1 stays inactive because of substrate inhibitions [313, 317]. The abundance of NMNAT2, the main axonal NAD^+^ provider, is tightly regulated by proteosome degradation and mitogen-activated protein kinase (MAPK) signaling [318, 319]. Upon axonal injury or mitochondrial insults, NMNAT2 level is reduced and subsequently SARM1 gets activated by the increased ratio of NMN (NMNAT2 substrate) to NAD^+^ [312, 320–323]. Activated SARM1 degrades NAD^+^ and leads to a rapid NAD^+^ decay, resulting in severe energetic failure in axons. It is yet to be determined whether NADP^+^ synthesis by NADK is required to suppress SARM1 activity and if SARM1 also mediated NADP^+^ degradation to exacerbate oxidative stress [324]. Regardless, constraining SARM1 activation seems to be the prerequisite for healthy bioenergetics in axons.

### Axonal bioenergetic maladaptation in aging and NDAs

The contribution of axonopathy and synaptopathy to functional decline during aging and the pre-symptomatic stage of NDAs has been extensively studied [60, 325–327]. Meta-analysis of 417 studies focusing on synaptopathy in postmortem AD brains reveals a consistent loss of synapses in hippocampus and frontal cortex, in which the presynaptic markers are more affected than the postsynaptic markers [328]. The previous sections of this review have summarized the subdomain-specific organization and regulation of the axonal bioenergetic system that crucially fuels axonal functions. In this section, we will discuss how axonal bioenergetics become altered during aging and NDAs. We first provide a phenotypic overview of axonal bioenergetic maladaptation upon aging and its progressive dysfunction since early-stage NDAs. Second, we will review the following signature aspects in aging/NDAs brains: cortical network hyperexcitability, NAD^+^ redox homeostasis disruption, and pathological glial responses. We also speculate how these different dysfunctions may further accelerate the failure of axonal bioenergetics.

#### Phenotypic overview

Increasing literature provides phenotypic evidence at subcellular resolution for aging-induced mitochondrial maladaptation in axons. In myelinated axons of the mouse optic nerve, aging results in reduced axon number, enlarged axon diameter, thickened myelin sheath, and increased nodal/paranodal length [329]. Within these aged optic nerve axons, mitochondrial density, and mitochondria-smooth endoplasmic reticulum contacting area are decreased, while the volume and length of individual mitochondrion are significantly increased [329]. Similar aging induced mitochondrial morphology and distribution changes were also observed in myelinated axons projecting to hippocampal CA1 and dentate gyrus regions [73]. In *C. elegans*, in vivo analysis of mitochondria in distal neurites throughout adult life reveals a progressive decline in mitochondrial trafficking starting from early adulthood, with mitochondrial size, density, and resistance to oxidative stress undergoing three distinct stages of increase, maintenance and decrease [330]. Reduced mitochondrial transport is also observed in *Drosophila* wing neuron axons [78, 79], and mouse retina nerve fiber layer myelinated axons [80] during aging.

In presynaptic boutons, aging elicits region-divergent adjustments of mitochondrial contents. Ultrastructural analysis of the hippocampal CA1 area prepared from male rats found significantly reduced numbers of mitochondria per presynaptic bouton, accompanied by an increased individual mitochondrion area in aged rats compared to adolescent and adult rats [331]. These synaptic mitochondria in aged synapses also exhibit functional impairment, including decreased Ca^2+^ buffering capacity, reduced ATP production, and increased oxidative stress [332–334]. Conversely, in the central nucleus of amygdala of aged rats, a brain region less affected by aging and neurodegenerative conditions, the number of mitochondria per bouton is drastically increased, while individual mitochondrion area is decreased compared to adult rats [335]. Similarly, in dorsolateral prefrontal cortex (dlPFC) of female *Rhesus* monkeys, studies found more mitochondria-containing boutons in aged peri/postmenopausal monkeys than in young and aged premenopausal moneys, with no difference in bouton densities or sizes [220]. Surgical menopause, causing abrupt estrogen loss, results in an increase of boutons containing donut shaped mitochondria and small active zones, and worsened working memory [220]. This finding suggests sexually dimorphic modulation of mitochondria and energy metabolism, a topic being widely reviewed [336–338]. In synaptosomal mitochondria samples purified from the whole brain, aging elicits mitochondrial DNA (mtDNA) deletion and activation of nuclear respiratory factor 1 (NRF1) and peroxisome proliferator-activated receptor gamma coactivator 1 alpha (PGC1A) mediated mitochondrial fitness pathways, accompanied by the adaptive increase of proteins governing OXPHO activity, antioxidant capacity, mitochondrial fusion and mitophagy [339]. These adaptive changes likely reduce the damage caused by mtDNA deletion, preserving mitochondrial respiration in aged brains [339]. Collectively, diverse mitochondrial alterations in myelinated and presynaptic compartments have been associated with aging.

Mitochondrial malfunction significantly worsens in both axonal and presynaptic compartments as NDAs progress. For example, EM studies found a substantial reduction in the density of mitochondria-resident presynapses in AD postmortem auditory association cortex of the temporal lobe, a DMN hub, compared to controls [340]. Interestingly, mitochondrial abnormalities were not detected in the dorsolateral prefrontal cortex, which is not involved in the DMN. A follow-up study from the same group found significantly less OXPHO machinery, but more Sirtuin pathway components, in synaptoneurosomes prepared from the auditory association cortex of AD brains, compared to controls, while no changes were found in the primary visual cortex [341]. Additionally, proteomic profiling of synaptoneurosomes prepared from parietal association cortex from MCI and dementia patients shows a synaptic metabolic shift towards glycolysis, while synaptic metabolism was rewired towards enhanced OXPHO in cognitively normal and resilient individuals [342]. A shift towards glycolysis can be interpreted as attempted compensation for mitochondrial dysfunction. Overall, the above evidence suggests a strong correlation between synaptic mitochondrial dysfunction and presynaptic degeneration, particularly in AD vulnerable brain regions.

Lessons from transgenic AD mouse models mimicking human amyloidogenesis suggest synaptic, but not non-synaptic mitochondria, are defective at pre-symptomatic stages, with defects including decreased mitochondrial respiration, compromised Ca^2+^ buffering, increased oxidative stress, increased permeability, elevated fission, and activation of Parkin-mediated mitophagy [103, 343–345]. A more detailed summary of synaptosomal mitochondrial deficits in current AD mouse models can be found in [345]. Resembling the presynaptic degeneration in AD brains, a preferential loss of excitatory synapses lacking presynaptic mitochondria was observed in the medial prefrontal cortex of pre-symptomatic 5xFAD mice [346]. Additionally, the mitochondrial quantity per presynaptic bouton was significantly reduced in the remaining mitochondria-containing synapses, perhaps due to mitophagy [346].

In postmortem brains of patients with PD and dementia with Lewy bodies (DLB), a similar presynaptic degeneration phenomenon occurs in substantia nigra (SN) dopaminergic neurons, in which the population of presynapses devoid of mitochondria is significantly reduced, despite the increase of mitochondrial proteins within the surviving dopaminergic axons [347]. In addition, the translation of mitochondrial proteins in synapses is dysregulated, revealed by proteomic characterization of synaptosomes from SN pars compacta (SNpc) of human PD brains [348]. Synaptosomal proteomic analysis with SNpc prepared from a PD mouse model overexpressing mutant human α-synuclein further sheds light on the importance of synaptic metabolic rewiring that precedes mitochondrial damage and synaptic pathology [114, 349]. In the mid-stage of this PD model, striatal synaptosomal mitochondria start to exhibit bioenergetic deficits, while no change in the abundance of essential OXPHO components and mitochondrial ultrastructure was detected [114]. Evidence suggests the functional decline could arise from the compromised proteostasis of antioxidant machineries. Overexpressed α-synuclein accumulates in presynaptic sites, interacts with the mitochondrial chaperone heat shock protein 10 (HSP10), and prevents its import into mitochondria, resulting in compromised quality control of essential antioxidant machineries within the mitochondrial matrix which eventually leads to synaptic mitochondrial dysfunction [350].

During the pre-symptomatic stage of HD mouse brains, the mitochondrial protein import deficit also occurs exclusively in the synaptosome, preceding any observable OXPHO impairment [351]. Simultaneously, striatal astrocytes in HD mouse brains acquire a metabolic adaptation towards fatty acid oxidation to compensate for diminished glucose uptake [352]. This leads to subsequent exposure of substantial astrocyte-derived reactive oxygen species which exacerbates the synaptic mitochondrial homeostasis disruption discussed above [352]. Likewise, in an ALS mouse model, defective mitochondrial respiration occurs in presynaptic boutons in the spinal cord at a pre-symptomatic stage without changes in OXPHO machinery abundance and mitochondrial quantity [353]. This study observed an increase in upstream glucose catabolism activity, including glycolysis and TCA cycle, as well as the lipid peroxidation in ALS mouse presynapses [353, 354]. In ALS mouse gliosomes, peri-synaptic astrocytic processes, lipid peroxidation and lactate fermentation were elevated, while mitochondrial respiration were preserved [353, 354].

Consistent across these major NDAs, metabolic alteration and mitochondrial dysfunction arise in the presynaptic compartment, representing the initial metabolic defects in the pre-symptomatic stage. They then interact with a reprogrammed glial environment in a negative cycle and eventually lead to synaptic connectivity deterioration and cognitive decline. Many of the current NDA animal models were generated based on the hypothesis that toxic misfolded protein aggregates are at the root of disease progression. For instance, the amyloid cascade hypothesis has been popular for decades in explaining AD, however the negative clinical outcomes to amyloid-directed therapies call for consideration of alternative hypothesis and models [355]. Here we want to endorse the alternative hypothesis that metabolic failure originating from the presynaptic compartment is one of major causes of neurodegeneration that culminates in cognitive function decline. We call for basic research with novel mouse disease models, covering a broad panel of late-onset NDAs as well as human brain organoid and co-culture system to test this hypothesis. In clinical aspects, we call for the development of multimodal biomarkers to infer the early-onset metabolic alterations in human brains, followed by longitudinal surveillance to elucidate its correlation with disease progression.

#### Network hyperexcitability

Partly due to reduced GABAergic tone [356–358], cortical network hyperexcitability increases during normal aging with excessive APs and increased synaptic transmission, which requires an extravagant energy demand, potentially overwhelming axonal bioenergetic capacity and triggering maladaptations. As axon length increases, the mitochondrial membrane potential declines [222], decreasing energy availability in the distal portion of long-range axons and providing less bioenergetic support for hyperexcitability. In addition, mitochondrial protein turnover is likely to be reduced as axon length increases [221, 222], posing additional difficulties on repairing and replenishing exhausted mitochondria in the synaptic boutons of distal axons.

Developing into the asymptomatic phase of NDAs, network hyperexcitability is further augmented before reaching the ultimate degeneration stage. In the prodromal phase of AD, the resting state activity is aberrantly increased in the medial prefrontal cortex, posterior cingulate cortex, precuneus, and hippocampus—all are connection hubs of DMN manifesting glucose hypometabolism [359, 360]. In apolipoprotein E4 (APOE-ɛ4) allele carriers, the strongest genetic factor for late-onset AD, lateral parietal and precuneus regions of DMN in the right-hemisphere show hyperconnectivity and hyperactivity events developing from a young age of 25.2 ± 6.8 years old [361]. Computational modeling suggests microscale hyperexcitability can increase the power of the lower frequency oscillatory network [362, 363], which is frequently observed in AD patients [363] and may underlie cognitive dysfunction [364]. Pre-symptomatic neuronal hyperexcitability in disease-vulnerable brain regions has also been documented in PD [365, 366], HD [367], and ALS [368].

Even though the casual relationship between hyperexcitability and glucose hypometabolism remains elusive, we speculate the excessively elevated synaptic activity directly poses heavy burden onto axonal bioenergetic system and rapidly consumes NAD^+^. Furthermore, excitotoxicity triggered by excessive glutamate release and Ca^2+^ influx can activate a necroptosis pathway that is reported to non-canonically deplete axonal NMNAT2 and activate SARM1, which destabilizes NAD homeostasis, exacerbates glucose hypometabolism, and eventually drives axonal degeneration and network disintegration [369, 370].

#### NAD redox homeostasis disruption

**Fig. 3:**
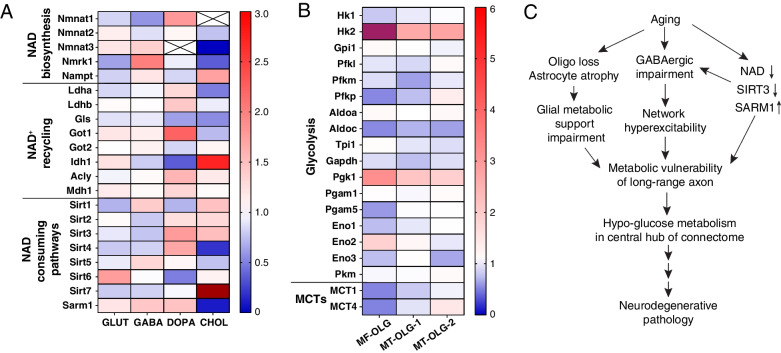
Aging induced bioenergetic maladaptation in mouse brain. **A**. Transcriptional fold change of enzymes involved in NAD^+^ redox potential maintenance in glutamatergic (GLUT), GABAergic (GABA), dopaminergic (DOPA) and cholinergic (CHOL) neurons upon aging, adapted from [22]. Sirt7 in aged cholinergic neurons is increased 5.97-fold, labeled in dark red. **B**. Transcriptional fold change of glycolytic enzymes and monocarboxylic acid transporters (MCTs) in myelin-forming oligodendrocytes (MF-OLG) and mature oligodendrocytes (MT-OLG). (MT-OLG-1 and MT-OLG-2 are two independent repeats in [22]). HK2 in aged myelin-forming oligodendrocytes is increased 9.72-fold, labeled in purple-red. **C**. A hypothetical model of the sequential events caused by aging that leads to metabolic failure in long-range axonal projections and glucose hypometabolism in central hubs of the brain connectome

NAD^+^ redox potentials decline in aged brains [371, 372]. The mRNA levels of several genes involved in NAD^+^ biosynthesis, NAD^+^ recycling and Sirtuin signaling are broadly altered in glutamatergic, GABAergic, dopaminergic, and cholinergic neurons during aging (Fig. 3A; [22]). For example, NMNAT2 mRNA level is decreased in both aged GABAergic and cholinergic neurons, while NMNAT1 mRNA level is reduced in aged glutamatergic and GABAergic neurons (Fig. 3A). Reduced NMNAT1/2 levels are likely to activate SARM1 to further reduce NAD^+^ levels and result in glucose hypometabolism in these neurons, particularly in GABAergic neurons. SIRT3 signaling has been shown to protect neuronal mitochondria against metabolic and excitatory stress [373]. However, SIRT3 mRNA levels are downregulated in both aged glutamatergic and GABAergic neurons (Fig. 3A). Thus, it is noteworthy that GABAergic neurons are particularly vulnerable to SIRT3 loss [374]. Reduced SIRT3 together with the NAD^+^ deficiency caused by NMNAT1/2 downregulation is likely to impair GABAergic glucose metabolism and subsequently increase hyperexcitability to further augment mitochondrial metabolic stress in axons.

Meta-analyses of human transcriptomic data found defective NAD metabolism in the majority of NDAs [375, 376]. In AD, NMNAT2 expression is significantly decreased in the frontal lobe [377, 378], while LDH-A mRNA level is reduced by almost twofold in both the frontal and temporal lobes [377]. Computational analysis with perturb-Met further predicts the dysregulation of NAD redox homeostasis in cortical layer 2–6 excitatory neurons of AD brains [379]. Similarly, dysregulated NAD homeostasis was also found in an AD mouse model, 3xTg transgenic mice, in a sex-biased manner [380, 381]. Noteworthy, assessed by two-photon lifetime imaging in dissociated neurons from mouse hippocampus of different ages, mitochondrial NADH level is significantly lower in 3xTg neurons, and declines with age [382]. However, due to the concern that cultures of dissociated adult brain neurons may not be disease relevant, more in vivo evidence is required to support this finding. Interestingly, a metabolomic study separating grey and white matter from postmortem brains found that nicotinamide metabolism-relevant metabolites (including NAD^+^,NADH, NMN, and nicotinamide) are significantly reduced in white matter compared to grey matter (supplementary table of [383]). This finding implies the myelinated compartment (that is, axons and their supporting cells) might be more susceptible to NAD decline. Using preclinical NDA models, numerous studies provide strong evidence supporting the therapeutic benefits of supplementing NAD^+^ and its precursors [384, 385]. The maintenance of NAD redox potentials is likely to enable optimal axonal bioenergetics and thus protect brain circuits.

To counteract the NAD redox disruption in NDAs, genetic approaches have been applied to enhance NAD salvage/biosynthesis pathways by overexpressing NMNATs, or to suppress NAD degradation by ablating SARM1. It has been shown that overexpressing NMNAT2 results in an increase in NAD redox potential in an AD transgenic cell line [386]. In the Tg2576 AD mouse model, NMNAT2 overexpression activates the AMPK signaling cascade, upregulates α-secretase expression, and attenuates β-secretase dependent amyloidogenesis [386]. The neuroprotective effect of different NMNAT isoforms on various neurodegenerative or neuropathy models has been summarized [387]. The neuroprotection against disease progression by NMNAT overexpression differs across disease models. There is substantial supportive evidence for neuroprotection offered by SARM1 deletion in animal models for traumatic brain injury and retinal degeneration [388–392]. However, there are also several reports showing minimal/no therapeutic benefits of SARM1 loss in PD [393] and ALS models [394, 395]. While the common downstream players of diverse NDAs converge at energy metabolism pathways, the upstream mechanisms are rather disease specific and complex. We hypothesize that targeting multiple upstream pathways that intersect with energy metabolism, together with stabilizing NAD homeostasis, should improve and prolong neuroprotection against NDAs. Recognizing the distinct excitability, energetic demands, mitochondrial regulatory units, proteome composition, and Ca^2+^ homeostasis in various neuronal subtypes [396, 397], we encourage the revisit of the axon protective effect offered by NMNAT2 and SARM1 manipulation in the most vulnerable neuronal subtypes in individual NDA models to dissect out the subtype specific mechanisms.

#### Deleterious glial dysfunction

Besides hyperexcitability and NAD^+^ redox perturbation, aging also elicits region-dependent alterations in glial biology [398–401]. For instance, oligodendrocyte differentiation is diminished in aged human brains, particularly prominent in the hippocampus and substantia nigra [399]. Additionally, oligodendrocyte numbers and myelin density are decreased in aged frontal cortex, concomitant with the appearance of myelin spheroids and debris [224, 399]. The reduced transcription of most glycolysis and MCTs genes in the oligodendrocytes of aged mouse brain (Fig. 3B) are likely to reduce their glucose metabolism capacity and decrease their metabolic support of myelinated axons.

Oligodendrocytes also undergo pathological changes as NDAs develop. Such glia alterations likely worsen glia-axon metabolic coupling and aggravates aging-induced maladaptation of axonal bioenergetics. The loss of myelination, oligodendrocytes, and OPCs has been frequently observed in the pre-symptomatic stage of AD and ALS animal models [402–405]. Consistently, in human postmortem AD brains, the number of mature myelinated oligodendrocytes is decreased, while remyelinating oligodendrocytes are increased [406]. Expanding on the fact that oligodendrocytes are inherently heterogeneous in the CNS [407], Sadick et al*.* further delineated the subtype-specific transcriptomic profile of oligodendrocytes in human AD brains. The biggest subcluster exhibiting downregulated expression were synaptic cell adhesion molecules, implying the loss of contact between oligodendrocytes and axons [408]. Interestingly, other subclusters show upregulation of putative neuroprotective pathways such as cholesterol metabolism [408], which accelerates Aβ production and in turn kills oligodendrocytes [409]. PD mouse model generated through alpha-synuclein injection into the dorsal striatum also showed the pathological inclusion of alpha-synuclein in oligodendrocytes secondary to neuronal pathology [410]. In iPSC-derived oligodendrocytes from PD patients, the transcriptional programs for maturation and myelination are significantly dampened and shifted towards immune reactive status, likely ascribed to the alpha-synuclein burden within oligodendrocytes [411]. Consistently, the population of myelinating oligodendrocyte is reduced in human PD postmortem midbrains, accompanied by the transcriptomic upregulation of unfolded protein response and downregulation of neuro-supportive pathways [412]. Future studies should be conducted to assess the metabolic decoupling between axons and oligodendrocytes in NDA brains and to determine whether such decoupling poses significant burdens to axonal bioenergetics. Furthermore, the remyelinating and immune reactive process in oligodendrocytes seem to be energy demanding [413, 414], potentially competing for the glucose metabolism substrates and therefore worsening the axonal bioenergetics.

Astrocytes have been shown to be altered in aging brain in a region-specific manner. Neurotoxic astrocyte subtypes found in aged mouse hippocampus and striatum exhibit downregulated genes in mitochondrial function, antioxidant defense, and cholesterol synthesis [400, 401]. The morphological complexity of astrocytes significantly decreases in AD-susceptible brain regions during aging, such as the entorhinal cortex and hippocampal CA1 region [402, 403]. Reduced astrocyte end-feet surface area is likely to diminish astrocytic coverage on synapses, and subsequently retard neurotransmitter uptake, further exacerbating neuronal hyperexcitability, while also impairing the astrocyte-neuron metabolic coupling that is essential for presynaptic bioenergetics. Similarly, as NDAs develop, astrocytes undergo substantial reprogramming in both morphological and metabolic aspects [415–420]. However, due to the evolutionarily divergent gene expression profiles, human astrocytes appear distinct from rodent astrocytes in Ca^2+^ signaling and metabolic activities [421, 422]. Such species-specific features raise the concern on whether astrocytic metabolic changes observed in AD mouse model are relevant to AD patients. For example, glycolytic and the mitochondrial TCA cycle activity are often reported to be decreased in astrocytes derived from AD mouse models [416]. However, the large-scale proteomic analysis of human AD postmortem brains suggests enhanced sugar metabolism in astrocytes and microglia [423]. Transcriptionally, astrocytes from human AD prefrontal cortex also exhibit remarkably differences from mouse 5xFAD cortex, featured by the downregulation of genes coordinating free-fatty-acid transport, lipid droplet storage and ROS detoxification [424]. In addition, the genes responsible for synaptogenesis and astrocyte morphogenesis are downregulated in certain subsets of astrocytes from AD brains, suggesting the trend towards tripartite synapse disintegration [408]. In late-stage PD patients, the interaction between astrocytes and excitatory neurons is estimated to be decreased by ~ 25% in the prefrontal cortex [425]. Given that astrocytic end-feet in tripartite synapses provide indispensable metabolic support, the astrocytic dysfunction mentioned above is anticipated to worsen the presynaptic bioenergetics that is already problematic since the pre-symptomatic stage. Overall, the characteristics and mechanism of glial metabolic alterations in human NDA brains remains largely unknown, as well as its relationship with axonal bioenergetic maladaptation. Therefore, we call for the development of multimodal biomarkers specific for glial metabolism to dissect out the region divergent changes and delineate the longitudinal profile during diseases progression, hoping to shape the direction for mechanistic study. Additionally, developing better experimental models recapitulating axon-glia interaction in human brains (e.g., human brain organoids) are important future directions.

In conclusion, we hypothesize (Fig. 3C) that network hyperexcitability occurs in disease-specific brain regions in NDAs, together with the NAD redox disruption, which triggers the axon-intrinsic metabolic failure that will be inherently determined by its bioenergetic supply system. As the disease progresses, glial alterations take place concomitantly in disease sensitive regions, leading to the destruction of the extrinsic metabolic support system that in turn accelerates the deterioration of axonal bioenergetics, synaptic connectivity, and cognitive function.

### Concluding remarks

Axons, key components of the brain connectome, are the most morphologically complex subcellular compartment in neurons and are prone to deterioration upon various insults. Aging, the most prevalent risk factor for many neurodegenerative diseases, is associated with decreased glucose metabolism, preferentially in distal axons and their terminals. This altered metabolism is accompanied by the mitochondrial maladaptation across myelinated axonal shaft and presynaptic boutons. These closely correlated phenotypes have driven the discovery of the molecular underpinnings that mediate age-associated bioenergetic incompetence and extrinsic glial metabolic support breakdown. Manipulating these molecular targets to enhance or normalize bioenergetics in specific cell types in NDA models will help to further interrogate the causal relationship between metabolic vulnerability, axonopathy, disease specific pathology and cognitive decline. Other than innate bioenergetic incompetency, additional efforts are needed to understand the influence of NDA relevant genetic, epigenetic, and environmental factors on neuronal bioenergetics.

## Data Availability

Not applicable.

## Abbreviations

3D: 3-Dimensional
ACLY: ATP citrate lyase
AD: Alzheimer’s disease
AIS: Axon initial segment
ALS: Amyotrophic lateral sclerosis
AMPK: AMP-activated protein kinase
AP: Action potential
APOE: Apolipoprotein E
APP: Amyloid precursor protein
ATP: Adenosine triphosphate
ATP5A1: ATP synthase F1 subunit alpha
ATP5B: ATP synthase F1 subunit beta
BACE1: Beta-secretase 1
C9ORF72: Chromosome 9 open reading frame 72
CNS: Central nervous system
COX IV: Cytochrome c oxidase subunit 4
cx-DHED: Carboxy-dehydroevodiamine∙HCl
DLB: Dementia with Lewy bodies
DMN: Default mode network
Drp1: Dynamin-related protein 1
DTI: Diffusion tensor imaging
DVR: Delayed verbal recall task
EM: Electron microscopy
FADH_2_: Oxidized flavin adenine dinucleotide
FDG-PET: Fluorodeoxyglucose-positron emission tomography
FTLD: Frontotemporal lobar degeneration.
GLP-1: Glucagon-like peptide 1
GLS: Glutaminase
GLUT: Glucose transport
GOT: Glutamic-oxaloacetic transaminase
GTP: Guanosine triphosphate
HCAR1: Hydroxycarboxylic acid receptor 1
HD: Huntington’s disease
HSP10: Heat shock protein 10
IDH1: Isocitrate dehydrogenase (NADP +) 1
iPSC: Induced pluripotent stem cells
LDH-A: Lactate dehydrogenase isoform A
LDH-B: Lactate dehydrogenase isoform B
LRRK2: Leucine-rich repeat kinase 2
LTP: Long-term potentiation
MAPK: Mitogen-activated protein kinase
MCI: Mild-cognitive impairment
MCT: Monocarboxylic acid transporters
MDH1: Malate dehydrogenase 1
MMSE: Mini Mental State Examination
mPTP: Mitochondrial permeability transition pore
MRI: Magnetic resonance imaging
mtCB_1_: Mitochondrial type-1 cannabinoid receptor
mtDNA: Mitochondrial DNA
NAD: Nicotinamide adenine dinucleotide
NADH: Oxidized nicotinamide adenine dinucleotide
NADK: NAD^+^ kinase
NAMPT: Nicotinamide phosphoribosyl-transferase
NDA: Neurodegenerative disorders of aging
NDUFS8: NADH:ubiquinone oxidoreductase core subunit S8
NMJ: Neuromuscular junction
NMN: Nicotinamide mononucleotide
NMNAT: Nicotinamide mononucleotide adenylyl transferase
NRF1: Nuclear respiratory factor 1
O-GlcNAcylation: O-linked-N-acetylglucosaminylation
OPC: Oligodendrocyte precursor cell
OSCP: ATP synthase peripheral stalk subunit OSCP (ATP5PO)
OXPHO: Oxidative phosphorylation
PAK: P21-activated kinase
PARP: Poly (adenosine diphosphate-ribose) polymerase
PCC: Posterior cingulate cortex
PD: Parkinson’s disease
PDH-E1α: Pyruvate dehydrogenase E1 subunit alpha 1
PFKFB3: 6-Phosphofructo-2-kinase/fructose-2,6-bisphosphatase-3
PGC1A: Peroxisome proliferator-activated receptor gamma coactivator 1 alpha
PiB: Amyloid-beta biomarker
PPARγ: Peroxisome proliferator-activated receptor gamma
PSEN1/2: Presenilin1/2
SARM1: Sterile alpha and TIR motif containing protein 1
SIRT: Sirtuin
SN: Substantia nigra
SNpc: SN pars compacta
STED: Stimulated emission depletion
SV: Synaptic vesicle
TCA: Tricarboxylic acid
α-Syn: α-Synuclein
